# Suppression of the toxicity of Bac7 (1–35), a bovine peptide antibiotic, and its production in *E. coli*

**DOI:** 10.1186/s13568-016-0190-3

**Published:** 2016-03-02

**Authors:** Yojiro Ishida, Masayori Inouye

**Affiliations:** Department of Biochemistry and Cell Biology, Center for Advanced Biotechnology and Medicine, Rutgers-Robert Wood Johnson Medical School, 679 Hoes Lane West, Piscataway, NJ 08854 USA

**Keywords:** Antimicrobial peptide, Bovine, Bac7 (1–35), *E. coli*, Protein S, SPP system

## Abstract

Bac7 (1–35) is an Arg- and Pro-rich peptide antibiotic, produced in bovine cells to protect them from microbial infection. It has been demonstrated to inhibit the protein synthesis in *E. coli*, leading to cell death. Because of its toxicity, no cost effective methods have been developed for Bac7 production in *Escherichia coli* for its potential clinical use. Here, we found a method to suppress Bac7 (1–35) toxicity in *E. coli* to establish its high expression system, in which Bac7 (1–35) was fused to the C-terminal end of protein S, a major spore-coat protein from Myxococcus xanthus, using a linker containing a Factor Xa cleavage site. The resulting His_6_-PrS_2_-Bac7 (1–35) (PrS_2_ is consisted of two N-terminal half domains of protein S connected in tandem) was well expressed using the Single-Protein Production (SPP) system at low temperature and subsequently purified in a single step by using a Ni column. The combination of protein S fusion and its expression in the SPP system at low temperature appeared to suppress Bac7 (1–35) toxicity. Both the purified His_6_-PrS_2_-Bac7 (1–35) and His_6_-PrS_2_-Bac7 (1–35) treated by Factor Xa were proven to be a potent inhibitor for cell-free protein synthesis.

## Introduction

Antimicrobial peptides (AMPs) consisting of 10–50 amino acid residues have been discovered from insects to mammals, specifically targeting against either Gram-negative bacteria or Gram-positive bacteria or both (Daher et al. 1988; Xi et al. 2014; Jayamani et al. 2015). Some of them are fungicidal. Some AMPs form helical structures through the membrane to cause cell lysis in a broad range of micro-organisms (Jamasbi et al. 2014; Li et al. 2015), while the others having very high contents of proline and arginine residues inhibit the functions of essential intracellular components (Tu et al. 2011; Roy et al. 2015). As recent emergence of highly antibiotic-resistant or multi-drug resistant pathogens, AMPs have become attractive alternatives for the treatment of patients. The use of AMPs is considered to be somehow less problematic than the use of conventional antibiotics as AMPs induce resistant strains in a much lower frequency (Zasloff 2002; Perron et al. 2006). Furthermore, the use of AMPs together with conventional antibiotics may have synergistic effects for therapeutic purpose. On the other hand, the disadvantages of AMPs are their cytotoxicity to the host, their instability in the cells, and the cost of their synthesis. Among AMPs, proline-rich antimicrobial peptides (PR-AMPs) have been isolated from mammalian neutrophils and from haemolymph of some invertebrate species (Anderson and Yu 2003; Treffers et al. 2005; Paulsen et al. 2013).

Bac7 (1–35) is a PR-AMP isolated from bovine and belongs to the cathelicidin family (Scocchi et al. 1997). The cathelicidins serve a critical role in mammalian innate immune defense against invasive bacterial infection (Zanetti 2004), and Bac7 (1–35) was found from bovine neutrophils together with cathelicidin as a antimicrobial peptides (Romeo et al. 1988; Gennaro et al. 1989). Later, Bac7 (1–35) was shown to inhibit DNA, RNA, and protein synthesis in *E. coli* (Mardirossian et al. 2014) after penetrating in the cells through SmbA, a peptide transporter (Mattiuzzo et al. 2007). Recently, Bac7 (1–35) was shown to bind to 70S ribosome resulting in inhibition of protein synthesis (Mardirossian et al. 2014). Despite the high toxicity to Gram-negative bacteria such as *E. coli*, *Klebsiella pneumoniae*, *Salmonella typhimurium*, and *Enterobacter cloacae* at 1–10 μM, Bac7 (1–35) has remarkably low cytotoxicity to the host mammalian cells (not toxic even at 50 μM) (Tomasinsig et al. 2006). Therefore, Bac7 (1–35) has been extensively studied because of its potential use for clinical application. A method to stabilize Bac7 (1–35) by PEGylation has been developed to reduce its renal clearance by which Bac7 (1–35) still retains its antibacterial activity as well as cell penetration activity (Benincasa et al. 2015).

For pharmaceutical applications, development of a method for an efficient AMP synthesis is important, but no successful expression system for Bac7 (1–35) has reported. So far, the production of some AMPs has been successfully carried out with a yeast system since these AMPs are not toxic to yeast (Jiménez et al. 2014; Wang et al. 2014; Mao et al. 2015). Furthermore, some AMPs have been produced using an *E. coli* system in combination with fusion tags such as thioredoxin (Feng et al. 2012) glutathione S-transferase (GST) (Feng et al. 2014), maltose-binding protein (MBP) (Velásquez et al. 2011) and small ubiquitin-like modifier like protein (SUMO) and subsequent cleavage of the AMPs form the fusion proteins by proteases such as thrombin, tobacco etch virus NIa protease, bovine coaglulation factor Xa, and enterokinase, which recognize only short, linear peptide sequences. However, if the AMP activity can be retained without cleavage from the fusion construct, it would be so much convenient for the toxicity assay of the AMPs. For this purpose, the SUMO technology has been successfully applied to many AMPs such as plectasin (Zhang et al. 2015), cathelicidin (Luan et al. 2014) and CM4 (Li et al. 2011).

In the present paper, we attempted to express Bac7 (1–35) in *E. coli* cells. Since Bac7 (1–35) is highly toxic to *E. coli*, it is essential to suppress its toxicity for its production. For this purpose, we tested two different protein tags, SUMO and protein S from Myxococcus xanthus to examine if the fusion tags could reduce the toxicity and enhance the expression of Bac7 (1–35). Protein S is a major spore-coat protein, which has been used as an effective fusion tag (Kobayashi et al. 2009). Protein S consists of 173 amino acids, which is composed of two homologous domains, the 92-residue N-terminal and the 81-residue C-terminal domains (Bagby et al. 1994). The expression vector, pCold-PST, contains two N-terminal domains (PrS_2_), repeated in tandem, to the C-terminal end of which a cloned protein or peptide is fused. pCold-PST vector has been shown to enhance the expression as well as the solubility of a cloned protein (Kobayashi et al. 2009). Protein S fused to a target protein has been shown not to severely affect the structure and function of the protein to be fused (Kobayashi et al. 2009, 2012). Since AMPs are toxic to the cells when expressed in *E. coli*, the suppression of toxicity to the cells possibly is essential for the production of AMPs. For this purpose, we attempted to use the Single-Protein Production (SPP) system for the production of PrS_2_-Bac7 (1–35), in which MazF, an ACA-specific endoribonuclease from *E. coli* is induced to eliminate almost all cellular mRNAs except for the mRNA for His_6_-PrS_2_-Bac7 (1–35) that is designed to have no ACA sequences without altering its amino acid sequence (Zhang et al. 2003; Suzuki et al. 2005, 2006). This enables us to produce only His_6_-PrS_2_-Bac7 (1–35) in *E. coli* cells without producing any other cellular proteins. Indeed, we were able to produce His_6_-PrS_2_-Bac7 (1–35) in a reasonable amount in *E. coli* cells, while with the SUMO tag, we were unable to express the protein.

## Materials and methods

### Construction of pColdPrS_2_Bac7 (1–35) vector and pColdSUMOBac7 (1–35)

A codon optimized ACA-less Bac7 (1–35) gene (cgtcgtattcgtccgcgtccaccgcgtctgccgcgtccgcgcccgcgtccactgccgttcccacgtccaggtccgcgtccgattccacgtccgctgccattcccgtaa) was synthesized (IDT) and cloned into ACA-less pColdPrS_2_ (Takara Bio) by using an infusion cloning system (Clontech), generating pColdPrS_2_-Bac7 (1–35), which is capable to produce His_6_-PrS_2_-Bac7 (1–35). PrS_2_ consists of two N-terminal half domains of protein S repeated in tandem (Kobayashi et al. 2009, 2012). His_6_-PrS_2_ and Bac7 (1–35) was linked with a tetra peptide, Ile-Glu-Gly-Arg as the Factor Xa cleavage site. Factor Xa cleaves the peptide after Arg so that intact Bac7 (1–35) is released after Factor Xa treatment without any extra amino acid residues attached.

The codon-optimized ACA-less SUMO-Bac7 (1–35) gene was synthesized (IDT) and cloned into pColdII (Takara Bio) by using infusion cloning system (Clontech), generating pColdSUMOBac7 (1–35) vector. In order to produce Bac7 (1–35) as a fusion protein, BL21(DE3) cells transformed with either pColdPrS_2_-Bac7 (1–35) or pColdSUMO-Bac7 (1–35) were inoculated into 10 ml of LB medium and the culture was incubated at 37 °C. When OD_600_ reached 0.8, the culture was transferred to 15 °C and the fusion proteins were induced by the addition of 1 mM IPTG. The mixture was further incubated for overnight.

### Production and purification of His_6_-PrS_2_-Bac7 (1–35) using SPP system

BL21(DE3) co-transformed with pACYCmazF and pColdPrS_2_-Bac7 (1–35) was inoculated into 1 l of LB medium and the culture was incubated at 37 °C. When the OD_600_ reached 0.8, the culture was chilled on ice for 5 min, followed by incubation at 15 °C for another 1 h. Subsequently, 1 mM IPTG was added to induced MazF and the culture was incubated at 15 °C for overnight (Suzuki et al. 2006). The cells were collected by centrifugation and re-suspended into 20 ml of binding/washing buffer consisting of 20 mM Tris–HCl (pH 8.0), 500 mM NaCl, and 20 mM imidazole–HCl (pH 8.0). After breaking the cells by using a French press, the unbroken cells were removed by centrifugation at 14,000 rpm for 20 min. The supernatant thus obtained was subjected to further centrifugation at 50,000 rpm for 30 min to remove the membrane fraction. The supernatant fraction was mixed with 1 ml of Ni-resin equilibrated in binding/wash buffer and the mixture was incubated for 1 h at 4 °C. The Ni-resin was washed twice with 10 ml of washing buffer, and His_6_-PrS_2_-Bac7 (1–35) was eluted with 20 mM Tris–HCl (pH8), 500 mM NaCl and 300 mM imidazole–HCl (pH8.0). After collecting the eluted protein, the protein concentration was determined by the optical density at 280 nm using Nano Drop (Thermo Scientific), and the purity was examined by SDS-PAGE. After the protein fraction was dialyzed against 20 mM Tris–HCl (pH8.0) and 100 mM NaCl, it was concentrated to 3 mg/ml and stored at −80 °C.

### Cleavage by factor Xa and identification of Bac7 (1–35)

The 30 μg of His_6_-PrS_2_-Bac7 (1–35) was digested with 4 μg of factor Xa in a 50 μl mixture containing 20 mM Tris–HCl (pH 8.0). 50 mM NaCl and 2 mM CaCl_2_. The reaction mixture was then incubated for 4 h at 37 °C, and the cleavage product was analyzed by 19 % SDS-PAGE followed by Coomassie blue staining. In order to confirm Bac7 (1–35) by mass spectrometry, His_6_-PrS_2_-Bac7 (1–35) was cleaved by factor Xa protease and the reaction mixture was diluted 25 times by the matrix solution containing sinapinic acid (10 mg/ml) in 0.1 % trifloroacetic acid and 50 % acetonitrile) and spotted on to a target plate (Opti-TOF 384 well insert, ABSciex) and air dried, followed by mass spectrometric analysis by a MALDI-TOF (4800 MALDI-TOF/TOF, ABSciex) using the positive mid-mass linear mode from 2 to 30 kDa.

### Small scale purification of Bac7 (1–35) by ion-exchange column chromatography

Three hundred sixty μg of His_6_-PrS_2_-Bac7 (1–35) was digested with 5 μg of factor Xa in a 500-μl of 20 mM Tris–HCl (pH 8.0) containing 50 mM NaCl and 2 mM CaCl_2_. The reaction mixture was incubated for overnight at room temperature, and the cleaved Bac7 (1–35) was purified from the reaction mixture by ion-exchange chromatography using SP-Sepharose (GE healthcare). The column was equilibrated with 20 mM Tris–HCl (pH 8.0) and washed with 20 mM Tris–HCl (pH 8.0) containing 100 mM NaCl. Bac7 (1–35) was eluted with 20 mM Tris–HCl (pH 8.0) containing 1 M NaCl. All eluted fractions were collected and the Bac7 (1–35) concentration was determined at 595 nm with use of Pierce Coomassie Plus (Thermo Fisher Scientific) (Bradford 1976).

### Synthesis of Bac7 (1–16)

Bac7 (1–16), the N-terminal fragment from residue 1 to residue 16 of Bac7 (1–35), consists of Arg–Arg-Ile-Arg-Pro-Arg-Pro–Pro-Arg-Leu-Pro-Arg-Pro-Arg-Pro-Arg, which has been shown to still retain one-fourth of the Bac7 toxicity and inhibit protein synthesis (Benincasa et al. 2004; Seefeldt et al. 2016). This peptide was commercially synthesized (GenScript) and dissolved in 1× PBS to make a 0.2 mM stock solution.

### In vitro translation inhibition assay

PURExress In Vitro Protein Synthesis kit (New England BioLabs) was used in this study. The gene for dihydrofolate reductase (DHFR) was used as a positive control. The reaction mixture containing buffer A and buffer B supplied from NEB were mixed with 20 U of RNase inhibitor (Roche), linearlized DNA (10 ng/μl) and synthetic peptide Bac7 (1–16) (10 μM) or His_6_-PrS_2_-Bac7 (1–35) (10 μM) or water, and the reaction mixture was incubated for 2 h at 37 °C. Protein production was examined by SDS-PAGE followed by Coomassie blue staining.

### Growth inhibition test when His_6_-PrS_2_-Bac7 (1–35) is induced in *E. coli*

The *E. coli* strain, BL21(DE3) harboring either pColdPrS_2_ or pColdPrS_2_-Bac7 (1–35) was grown in the M9-glucose medium. When the OD_600_ reached at 0.2, 1 mM IPTG was added into the medium to induce the protein. As a negative control, the culture medium in the absence of IPTG was also incubated and the OD_600_ was monitored every 30 min.

### The antimicrobial activity of purified Bac7 (1–35) in *E. coli*

The *E. coli* strain BL21 (DE3) was grown in the M9-glucose medium and purified Bac7 (1–35) was added at the final concentration of 2 μM into the medium when OD_600_ reached 0.2. OD_600_ was monitored every 30 min.

## Results

### The expression and purification of His_6_-PrS_2_-Bac7 (1–35)

**Fig. 1:**
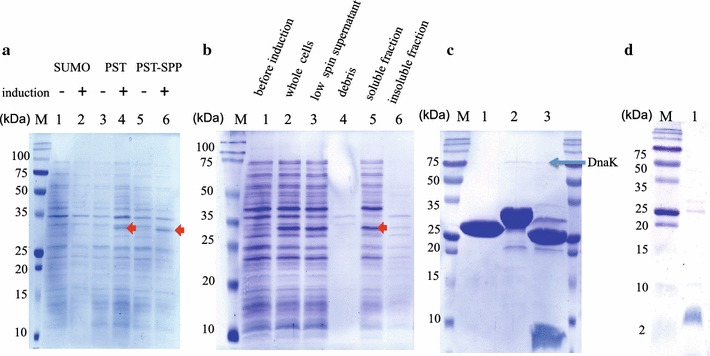
The production of SUMO-Bac7 (1–35) and PrS_2_-Bac7 (1–35) in *E. coli* BL21 (DE3). **a** BL21 (DE3) harboring either pColdSUMO-Bac7 (1–35) or pColdPrS_2_-Bac7 (1–35) was inoculated in the 5 ml of LB medium. After OD_600_ reached at 0.8, the 1 mM IPTG was added, and the culture was incubated overnight incubation at 16 °C. The position of PrS_2_-Bac7 (1–35) is indicated by an arrow. BL21 (DE3) co-transformed with pACYCmazF and pColdPrS_2_-Bac7 (1–35) (SPP cells) was inoculated into 5 ml of LB medium and the culture was incubated at 37 °C. Wen the OD_600_ reached at 0.8, the culture was transferred onto ice for 5 min, followed by 1 h incubation at 16 °C. After protein induction by the addition of 1 mM IPTG, the culture medium was incubated for overnight at 16 °C. *Lane 1*, SUMO-Bac7 (1–35) before induction; *lane 2*, SUMO-Bac7 (1–35) after induction; *lane 3*, PrS_2_-Bac7 (1–35) before induction; *lane 4*, PrS_2_-Bac7 (1–35) after induction; *lane 5*, SPP cells before induction; and *lane 6*, SPP cells after induction. **b** The PrS_2_-Bac7 (1–35) production using the SPP system and its cellular localization. The ACA-less gene for Bac7 (1–35) was expressed using pCold-PrS_2_ vector together with pACYCmazF at 15 °C. *Lane 1*, before 1 mM IPTG was added. After inducing the PrS_2_-Bac7 (1–35) at 15 °C for overnight, the cells was collected by centrifugation and subsequently cellular fractionation was carried out; *lane 2*, whole cells; *lane 3*, cell lysate after low speed centrifugation; *lane 4*, cell pellets after low speed centrifugation; *lane 5*, the soluble fraction, and lane 6, the insoluble fraction. **c** Cleavage of PrS_2_-Bac7 (1–35) by Factor Xa protease. The 30 μg of purified PrS_2_-Bac7 (1–35) in 50 μl was incubated with 4 μl of factor Xa protease at 37 °C for 4 h. After incubation, 10 μl of the reaction mixture was subjected to 20 % SDS-PAGE. *Lane 1*, PrS_2_ only; *lane 2*, PrS-Bac7 (1–35) before cleavage by factor Xa; *lane 3*, PrS_2_-Bac7 (1–35) after cleavage by Factor Xa; and *lane M*, molecular weight markers. DnaK, one of the target proteins of Bac7 (1–35), was co-purified, and shown by an *arrow.* **d** Bac7 (1–35) purified after from PrS-Bac7 (1–35) treated by factor Xa followed by ion-exchange column chromatography. The concentration of purified Bac7 (1–35) was determined by Bradford reagent, and 1.6 μg of Bac7 (1–35) was analyzed by 20 % SDS-PAGE

Since Bac7 (1–35) is highly toxic to *E. coli* cells, we attempted to express Bac7 (1–35) fused to the C-terminal end of protein S or SUMO at 15 °C. As a result, we were not able to observe the production of His_6_-SUMO-Bac7 (1–35), while His_6_-PrS_2_-Bac7 (1–35) was produced as detected at around 30-kDa position in SDS-PAGE gel (Fig. 1a). To further improve the expression of His_6_-PrS_2_-Bac7 (1–35), we attempted to apply the SPP system for its production. Since the SPP system allows one to produce only a target protein without producing any cellular proteins, it may help to produce His_6_-PrS_2_-Bac7 (1–35) to a better yield. As shown in Fig. 1a, the use of the SPP system indeed enhanced the production of His_6_-PrS_2_-Bac7 (1–35). Notable the use of the SPP system for the production His_6_-SUMO-Bac7 (1–35) was unsuccessful, probably because the SUMO tag could not suppress the toxicity of His_6_-PrS_2_-Bac7 (1–35).

After fractionation by ultracentrifuge, His_6_-PrS_2_-Bac7 (1–35) was fully recovered in the soluble fraction (Fig. 1b). The final yield after purification using Ni–NTA column chromatography was determined by a Nano Drop spectrophotometer to be 2.5 mg from 1 l LB medium. Higher than 90 % purification was achieved by one-step Ni–NTA purification (Fig. 1b).

### Purification of Bac7 (1–35) from His_6_-PrS_2_-Bac7 (1–35)

After treating His_6_-PrS_2_-Bac7 (1–35) with factor Xa, cleaved Bac7 (1–35) was purified by ion-exchange column chromatography. Since the pI value of His_6_-PrS_2_ is 5.75 while the pI value of Bac7 (1–35) is 13.0, His_6_-PrS_2_ and Bac7 (1–35) were readily separated by SP Sepharose. In addition, the sizes of His_6_-PrS_2_ and Bac6(1–35) are 21 and 4.2 kDa, respectively, so that the size of Bac7 (1–35) is about one-sixth of the fusion protein. Fifty-four μg of highly purified Bac7 (1–35) was obtained from 360 μg of the fusion protein, which was about 90 % yield (Fig. 1d). Since 2.5 mg of His_6_-PrS_2_-Bac7 (1–35) was obtained from 1 l LB medium, the estimated yield of Bac7 (1–35) was 0.36 mg.

### Identification of the Bac7 (1–35) fragment in His_6_-PrS_2_-Bac7 (1–35)

**Fig. 2:**
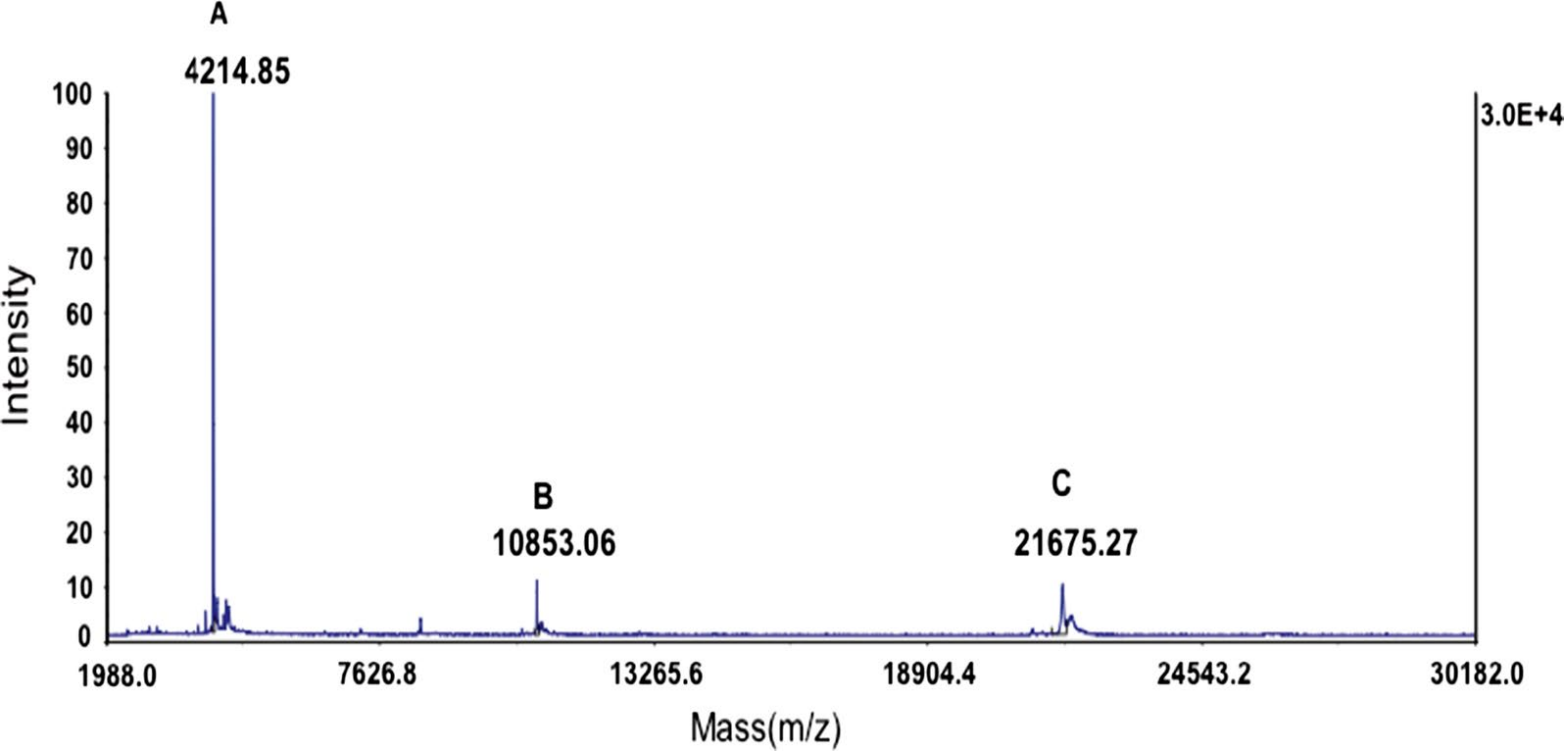
Molecular weight analysis using mass spectrometry. His_6_-PrS_2_-Bac7 (1–35) was cleaved by factor Xa protease and the reaction mixture was diluted 25 times by the matrix solution containing sinapinic acid (10 mg/ml) in 0.1 % trifloroacetic acid and 50 % acetonitrile) and spotted on to a target plate (Opti-TOF 384 well insert, ABSciex) and air dried, followed by mass spectroscopic analysis by a MALDI-TOF using the positive mid-mass linear mode from 2 to 30 kDa. *Peak A* (4.21 kDa) represents Bac7 (1–35) fragment, *peak B* (10.85 kDa) represents the PrS fragment, and *peak C* (21.68 kDa) represents the PrS_2_ fragment

In order to confirm the existence of the Bac7 (1–35) fragment in His_6_-PrS_2_-Bac7 (1–35), His_6_-PrS_2_-Bac7 (1–35) thus produced was treated with Factor Xa protease (Fig. 1c), and the digest was analyzed by SDS-PAGE followed by mass spectrometric analysis (Fig. 2). Note that since Bac7 (1–35) was fused to His_6_-PrS_2_ with a tetra-peptide linker, Ile-Glu-Gly-Arg, the treatment of the His_6_-PrS_2_-Bac7 (1–35) with Factor Xa releases the intact Bac7 (1–35). The molecular weights (MW) of PrS_2_ tag and Bac7 (1–35) are 21.5 and 4.2 kDa, respectively. The theoretical values of the MW for Bac7 (1–35) is calculated to be 4207 Da, agreeing well with the MW observed. The peak at 10.8 kDa was probably due to the initiation from the internal Met residue in the gene (Fig. 2).

### The function of His_6_-PrS_2_-Bac7 (1–35)

**Fig. 3:**
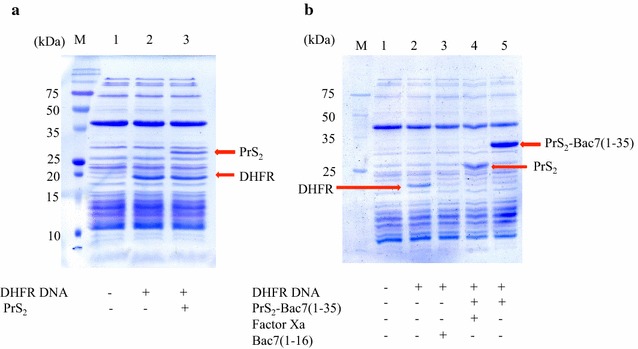
DHFR production using cell-free expression system. **a** DHFR expression in the presence or absence of PrS_2_. Solutions A and B were mixed according to the manufacturer’s protocol (NEB), and the mixture was incubated as a negative control (*lane 1*), and DNA(10 ng/μl) of DHFR were added and incubated as a positive control (*lane 2*), DNA of DHFR (10 ng/μl) together with 0.2 mg/ml of PrS_2_ (10 μM) were incubated. All incubations were performed at 37 °C for 2 h. After the incubation, 2× SDS loading dye was added and the mixture was subjected to the 17 % of SDS-PAGE and the molecular weight marker was shown as* lane M*; **b** DHFR expression in the presence of PrS_2_-Bac7 with and without factor Xa. Solutions A and B from the Cell-Free system (NEB) were mixed, the reaction mixture without DNA was used as a negative control (*lane 1*) and the reaction mixture in the presence of DNA (10 ng/μl) but in the absence of proteins was used as a positive control (*lane 2*). *Lane 3*, the reaction mixture containing DNA (10 ng/μl) together with 10 μM of Bac7 (1–16) as a control; *lane 4*, the reaction mixture containing DNA (10 ng/μl) together with 10 μM of PrS_2_-Bac7 (1–35) after cleavage by Factor Xa; *lane 5*, the reaction mixture containing DNA (10 ng/μl) together with 10 μM of PrS_2_-Bac7 (1–35) without cleaving by Factor Xa, and *lane M*, molecular weight marker. All the reactions were carried out at 37 °C for 2 h, After the reaction, 2× SDS loading buffer was added to the reaction mixture, which was then subjected to 15 % SDS-PAGE, followed by Coommassie blue staining

Although PST is known not to interfere the function of the fusion partner (Kobayashi et al. 2009), we next examined the inhibitory activity of the purified His_6_-PrS_2_-Bac7 (1–35) using a cell-free protein synthesis system (New England BioLabs) comparing with the inhibitory ability of intact fusion protein. As a positive control, the expression of dihydrofolate reductase (DHFR; 20 kDa) was examined in the absence and presence of PrS_2_. As shown in Fig. 3a, the addition of PrS_2_ did not have any effects on the protein synthesis. Next, the inhibitory effects were compared between His_6_-PrS_2_-Bac7 (1–35) and His_6_-PrS_2_-Bac7 (1–35) treated with Factor Xa. We also synthesized Bac7 (1–16), which was recently reported to inhibit protein synthesis (Seefeldt et al. 2016) and used as a positive control for the experiment. As shown in Fig. 3b, both His_6_-PrS_2_-Bac7 (1–35) and His_6_-PrS_2_-Bac7 (1–35) treated with Factor Xa inhibited the protein synthesis as well as Bac7 (1–16).

### The antimicrobial activity of His_6_-PrS_2_-Bac7 (1–35) in the cells

**Fig. 4:**
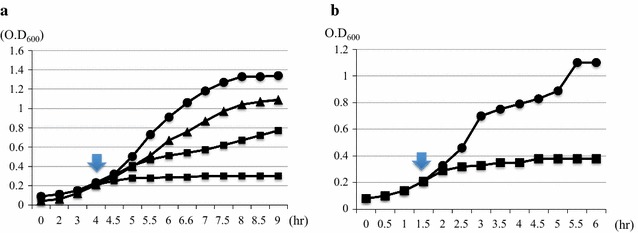
Toxicity of Bac7 (1–35) in *E. coli.* **a** The toxicity of pColdPrS_2_-Bac7 (1–35) in *E. coli*. BL21(DE3) harboring either pColdP_2_rS or pColdPrS_2_-Bac7 (1–35). The cells were grown in the M9 medium. When OD_600_ reached 0.2, PrS_2_-Bac7 (1–35) was induced by 1 mM IPTG, and OD_600_ was monitored every 30 min. The *circles* represent pColdPrS_2_ without IPTG, the triangles pColdPrS_2_-Bac7 (1–35) without IPTG, and the Xs pColdPrS_2_ in the presence of IPTG and the squares pColdPrS_2_-Bac7 (1–35). The time of the addition of IPTG is shown by an arrow. **b** The toxicity of purified Bac7 (1–35) in *E. coli*. The *E. coli* cells BL21(DE3) was grown in M9-glucose medium. When OD_600_ reached 0.2, purified Bac7 (1–35) was added at the final concentration of 2 μM into the medium, and OD_600_ was monitored every 30 min. The time of the addition of Bac7 (1–35) is shown by an *arrow*

Since His_6_-PrS_2_-Bac7 (1–35) was shown to retain the ribosome inhibition activity, we have tested the growth effect of induction of His_6_-PrS_2_-Bac7 (1–35) in the cells. As shown in Fig. 4a, the cell growth was totally arrested by His_6_-PrS_2_-Bac7 (1–35) after 30 min of induction, while the induction of His_6_-PrS_2_ did not cause the cell growth arrest.

### The antimicrobial activity of purified Bac7 (1–35) using *E. coli* cells

The purified Bac7 (1–35) was tested using *E. coli* cells. Bac7 (1–35) efficiently inhibited *E. coli* cell growth at 2 μM after 30 min (Fig. 4b).

## Discussion

**Fig. 5:**
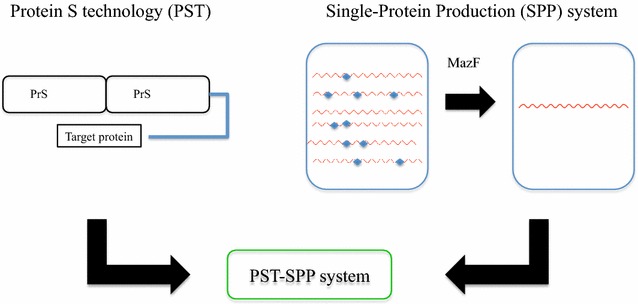
Schematic presentation of the PST-SPP system. PrS, a major spore-coat protein from *Myxococcus xanthus*, was directly repeated (PrS_2_) and used as a tag (PST tag) for higher expression and solubilization of a target protein. Functional assay for the target protein can be done without cleaving the PrS_2_ from the fusion protein. A codon optimized ACA-less gene for the target protein can be expressed together with pACYCmazF, cleaving the ACA sequences in mRNAs, allowing the cells to produce the only target gene from its ACA-less mRNA (the SPP system; Suzuki et al. 2005). Note that the gene for PrS_2_ is also codon-optimized for *E. coli* and designed to be ACA-less without altering the amino acid sequence. Since the PrS_2_ tag partially suppresses the antimicrobial activity of a cloned peptide or protein, the use of the PST-SPP system allows one to produce toxic peptides and proteins in *E. coli* and also to perform their functional assay using the cell lysate

The AMP production in *E. coli* is challenging because their antimicrobial activity. To suppress their toxicity, relatively large tags such as GST, MBP and SUMO may be used, however, for the most of AMP production, SUMO has been widely applied and many AMPs were successfully expressed as functional forms (Li et al. 2011; Zhang et al. 2014, 2015). The SUMO tag has been shown to improve protein folding and solubility, and to be used for protein detection (Luan et al. 2014). Thus, we attempted to examine if the fusion of Bac7 (1–35) to the C-terminal end of SUMO could suppress the Bac7 toxicity, but the production of the fusion protein was not detected, indicating that the SUMO tag could not suppress the toxicity of Bac7 (1–35), which is known to inhibit the function of 70S ribosomes (Mardirossian et al. 2014). Thus, we next tried protein S as a fusion tag for Bac7 (1–35). The Protein S from *Myxococcus xanthus* is known to function as an intra-molecular chaperone without severely affecting the function of the protein fused to it, and has been applied for the expression of proteins which are insoluble and/or difficult to be expressed (Kobayashi et al. 2009, 2012). In the present study, we used two 88-residue N-terminal domains repeated in tandem to the C-terminal end of which Bac7 (1–35) was fused. The resultant His_6_-PrS_2_-Bac7 (1–35) was indeed expressed well in the SPP system. In this PST-SPP system, an ACA-less gene encoding His_6_-PrS_2_ was used as an N-terminal tag for Bac7 (1–35) to produce His_6_-PrS_2_-Bac7 (1–35) (Fig. 5). We also constructed the ACA-less His_6_-SUMO-Bac7 (1–35) system. However, His_6_-PrS_2_-Bac7 (1–35) was expressed (Fig. 1a) while the expression of SUMO-Bac7 (1–35) was not detected, indicating that the SUMO tag was not able to suppress the Bac7 (1–35) toxicity even with use of the SPP system. Notably, however, the expression of His_6_-PrS_2_-Bac7 (1–35) was rather low, possibly because protein S fusion to Bac7 (1–35) did not completely suppress the toxicity of Bac7 (1–35). Indeed, pColdPrS_2_-Bac7 (1–35) in M9-glucose medium was toxic in the presence of 1 mM IPTG (Fig. 4a).

Bac7 (1–35) has been shown to inhibit the function of 70S ribosomes to block protein synthesis (Mardirossian et al. 2014). Thus, the activity of His_6_-PrS_2_-Bac7 (1–35) was tested using a cell-free protein expression system with a synthetic peptide Bac7 (1–16) as a protein synthesis inhibitor (Seefeldt et al. 2016) as a control. As shown Fig. 3b, the production of DHFR by the cell-free system was indeed inhibited by both His_6_-PrS_2_-Bac7 (1–35) and Bac7 (1–35) which was generated from His_6_-PrS_2_-Bac7 (1–35) by Factor Xa treatment which resulted in a small amount of uncleaved His_6_-PrS_2_-Bac7 (1–35) (Fig. 2c). Notably, the cleavage mixture effectively inhibited the protein synthesis (Fig. 3b). Since the minimum inhibitory concentration of Bac7 (1–35) has been reported to be 0.5 μM (Benincasa et al. 2004), it is assumed that there was an excessive amount of Bac7 (1–35) in the reaction mixture to inhibit protein synthesis. The PrS_2_ tag is known not to interfere with its fusion partner (Kobayashi et al. 2009); for example, PrS_2_ fused at the N-terminal end of OmpR, a phosphor sensory protein, did not inhibit the OmpR function at all (Kobayashi et al. 2009). Thus, it is not surprising to see that His_6_-PrS_2_-Bac7 (1–35) possesses an antibacterial activity in spite of the fact that the N-terminal part of Bac7 (1–35) has been shown to be crucial for the antimicrobial activity (Guida et al. 2015).

Using the SPP system, MazF cleaves at all ACA in mRNA while only the codon-optimized ACA-less gene for His_6_-PrS_2_-Bac7 (1–35) remains intact. Therefore, upon induction of MazF, only His_6_-PrS_2_-Bac7 (1–35) is produced in the cells (Fig. 5). Notably, in the SPP system, all the cellular mRNAs containing ACA sequences are digested by MazF, so that cell growth is completely arrested allowing the production of only the target protein from the ACA-less mRNA in the growth-arrested cells. In this manner, toxic proteins can still be produced as far as they do not inhibit ATP production and protein synthesis. Previously, we have demonstrated that it is possible to completely replace all arginine residues in a protein with canavanine, a highly toxic analogue of arginine using the SPP system, since the incorporation of canavanine into any other cellular proteins is well suppressed (Suzuki et al. 2006; Mao et al. 2009; Ishida et al. 2013). In the present paper, we combine both PST and SPP technologies (PST-SPP technology) to successfully express His_6_-PrS_2_-Bac7 (1–35).

In this study, we demonstrated to obtain 90 % pure His_6_-PrS_2_-Bac7 (1–35) by one-step purification. In addition, Bac7 (1–35) was readily purified from His_6_-PrS_2_-Bac7 (1–35) treated by factor Xa followed by ion exchange column chromatography using SP-Sepharose, since the pI value of His_6_-PrS_2_ is 5.75 while that of Bac7 (1–35) is 13.0. We were able to obtain highly pure Bac7 (1–35) with approximately 90 % yield. It is also important to note that since Bac7 (1–35) does not have any aromatic residues, the protein concentration should be determined by ninhydrin or the Bradford assay (Bradford 1976). While the chemical synthesis of long AMPs such as Bac7 (1–35) is highly expensive, the technology developed in the present paper will greatly reduce the cost of the AMP production.
